# Are *PARKIN* patients ideal candidates for dopaminergic cell replacement therapies?

**DOI:** 10.1111/ejn.14314

**Published:** 2019-01-23

**Authors:** Tilo Kunath, Ammar Natalwala, Claire Chan, Yixi Chen, Benjamin Stecher, Martin Taylor, Sadaquate Khan, Miratul M. K. Muqit

**Affiliations:** ^1^ MRC Centre for Regenerative Medicine Institute for Stem Cell Research School of Biological Sciences The University of Edinburgh Edinburgh UK; ^2^ Translational Neurosurgery Group Western General Hospital Edinburgh UK; ^3^ Tomorrow Edition Toronto Canada; ^4^ Edinburgh Research Interest Group Parkinson's UK Edinburgh UK; ^5^ MRC Protein Phosphorylation and Ubiquitylation Unit School of Life Sciences University of Dundee DD1 5EH Dundee UK

**Keywords:** dopaminergic cell transplantation, *PARKIN*, Parkinson's disease, pure nigropathy, synucleinopathy

## Abstract

Parkinson's is a heterogeneous, complex condition. Stratification of Parkinson's subtypes will be essential to identify those that will benefit most from a cell replacement therapy. Foetal mesencephalic grafts can alleviate motor symptoms in some Parkinson's patients. However, on‐going synucleinopathy results in the grafts eventually developing Lewy bodies, and they begin to fail. We propose that Parkinson's patients with *PARKIN* mutations may benefit most from a cell replacement therapy because (a) they often lack synucleinopathy, and (b) their neurodegeneration is often confined to the nigrostriatal pathway. While patients with *PARKIN* mutations exhibit clinical signs of Parkinson's, post‐mortem studies to date indicate the majority lack Lewy bodies suggesting the nigral dopaminergic neurons are lost in a cell autonomous manner independent of α‐synuclein mechanisms. Furthermore, these patients are usually younger, slow progressing and typically do not suffer from complex non‐nigral symptoms that are unlikely to be ameliorated by a cell replacement therapy. Transplantation of dopaminergic cells into the putamen of these patients will provide neurons with wild‐type PARKIN expression to re‐innervate the striatum. The focal nature of PARKIN‐mediated neurodegeneration and lack of active synucleinopathy in most young‐onset cases makes these patients ideal candidates for a dopaminergic cell replacement therapy. Strategies to improve the outcome of cell replacement therapies for sporadic Parkinson's include the use of adjunct therapeutics that target α‐synuclein spreading and the use of genetically engineered grafts that are resistant to synucleinopathy.

**Abbreviations**
AR‐JP: autosomal recessive juvenile Parkinson's
DAT: dopamine transporter
DAT‐SPECT: dopamine transporter‐single photon emission computed tomography
DLB: dementia with Lewy bodies
GBA: glucocerebrosidase
hESCs: human embryonic stem cells
iPSCs: induced pluripotent stem cells
LRRK2: leucine‐rich repeat kinase 2
mDA: midbrain dopaminergic
MIBG: metaiodobenzylguanidine
OMM: outer mitochondrial membrane
PD: Parkinson's disease
PFFs: pre‐formed fibrils
PINK1: PTEN induced kinase 1
pS129‐αSyn: phosphorylated serine‐129‐α‐synuclein
SNCA: synuclein, alpha gene
TH: tyrosine hydroxylase
VM: ventral mesencephalon

## 1 INTRODUCTION

Parkinson's disease (PD) is a common and complex neurodegenerative condition that has multiple underlying pathologies ultimately leading to disruption of the basal ganglia due to loss of dopaminergic innervation of the striatum from the substantia nigra. For over 30 years, transplantation of human foetal mesencephalic tissue into the striatum by stereotactic surgery has been attempted with mixed success (Lindvall et al., 1990, 1994; Olanow et al., 2003; Piccini et al., 2005). In some cases, the grafts restored dopamine transmission to near normal levels, reversed motor dysfunction and reduced dependence on dopaminergic medicines for at least 15 years (Barker, Barrett, Mason, & Björklund, 2013; Kefalopoulou et al., 2014). However, troublesome graft‐induced dyskinesias were common with up to 50% of patients experiencing this unexpected side‐effect (Hagell et al., 2002). This was caused in part by undesired serotonergic neurons present in the graft (Hagell et al., 2002; Politis et al., 2010). The problem of graft heterogeneity and unwanted cell types can be addressed by producing midbrain dopaminergic (mDA) neurons from human pluripotent stem cells that are highly pure and devoid of any serotonergic neurons (Kriks et al., 2011). The progress towards clinical trials for cell replacement therapies for Parkinson's is very advanced now (Barker, Parmar, Studer, & Takahashi, 2017), with the first Parkinson's patient transplanted in Japan in October 2018 (Cyranoski, 2018).

The pathological hallmark of PD, the Lewy body, is made up of aggregated proteins of which a major component is α‐synuclein (Spillantini et al., 1997). The pattern of Lewy body formation in distinct anatomical regions during disease progression has led to a prion‐like spreading hypothesis for PD with α‐synuclein proposed to be the prion‐like molecule (Braak, Ghebremedhin, Rüb, Bratzke, & Del Tredici, 2004). Further support for this hypothesis came when PD patients who had received foetal ventral mesencephalic grafts came to autopsy. The majority of grafts that were 10 years or older had clear signs of Lewy body formation and were exhibiting signs of decline, such as reduced dopamine transporter (DAT) expression (Kordower, Chu, Hauser, Freeman, & Olanow, 2008; Kordower, Chu, Hauser, Olanow, & Freeman, 2008; Li et al., 2008; Mendez et al., 2008). Foetal grafts that were 18 months or 4 years old showed little evidence of synucleinopathy, which suggests that if there is a host‐to‐graft transfer of Lewy pathology it is not particularly rapid (Chu & Kordower, 2010). Experimental evidence in support of this possible mechanism came from various models, including the stereotactic injection of recombinant pre‐formed fibrils (PFFs) of α‐synuclein into wild‐type mice (Luk et al., 2012). A time‐dependent spreading of Lewy‐like pathology was observed over 18 months, and this was dependent on the presence of the endogenous mouse *Snca* gene (Luk et al., 2012).

Although PD is usually sporadic, a significant number of cases (>10%) are familial (Hardy, Cai, Cookson, Gwinn‐Hardy, & Singleton, 2006). Point mutations in the *SNCA* gene encoding for α‐synuclein are a known cause of familial PD (Kiely et al., 2013; Krüger et al., 1998; Lesage et al., 2013; Pasanen et al., 2014; Polymeropoulos et al., 1997; Zarranz et al., 2004). Multiplications of the wild‐type *SNCA* gene are also an autosomal dominant cause of PD (Chartier‐Harlin et al., 2004; Ibáñez et al., 2004; Singleton et al., 2003). Genome‐wide association studies have also identified polymorphisms around the *SNCA* locus to be the most significant genetic risk factors for sporadic PD (Satake et al., 2009; Simón‐Sánchez et al., 2009). The most common mutations known to cause familial PD are autosomal dominant mutations in the *LRRK2* gene (Zimprich et al., 2004). The prevalence of the G2019S mutation of *LRRK2* in PD patient populations varies greatly, and has been found to be as high as 41% in the North African Berber sporadic PD population (Lesage et al., 2006). Most *LRRK2* patients have Lewy body pathology that is similar, if not identical, to sporadic PD (Santpere & Ferrer, 2009; Zimprich et al., 2004). However, there are accumulating reports of *LRRK2* patients with clinical PD and nigral degeneration, but without any evidence of Lewy body pathology (Gaig et al., 2007; Takanashi et al., 2018; Wszolek et al., 2004).

## 2 PURE NIGROPATHY PARKINSON'S DISEASE

Cases of Parkinson's without Lewy bodies began to appear in the literature in the early 1990s (Dwork et al., 1993). The patients were usually early‐onset (<40 years), slow progressing and showed a good response to Levodopa. This condition, distinct from sporadic PD, was often referred to as autosomal recessive juvenile Parkinson's (AR‐JP) and is prevalent in Japan (Yamamura, Sobue, Ando, Iida, & Yanagi, 1973). The underlying mutation found to cause AR‐JP was identified in the *PARKIN* gene (Kitada et al., 1998). Since this report, multiple families from across the world have been identified with diverse mutations in *PARKIN* (Cornejo‐Olivas et al., 2015; Farrer et al., 2001; Gouider‐Khouja et al., 2003; van de Warrenburg et al., 2001). The prevalence of *PARKIN* mutations in the young‐onset (<45 years) sporadic PD population has been estimated to be about 15% (Periquet et al., 2003), and this increases to almost 50% for familial young‐onset cases with a recessive pattern of inheritance (Bonifati, 2012; Lücking et al., 2000). Prior to the identification of *PARKIN* mutations, post‐mortem brain studies of Japanese patients who died with AR‐JP exhibited a striking lack of Lewy body pathology and very little neurodegeneration beyond the substantia nigra (Matsumine et al., 1997; Mori et al., 1998; Takahashi et al., 1994; Yamamura et al., 1998). Since these first reports, several *PARKIN* patients have been reported with α‐synuclein‐positive Lewy bodies, but they were often of older age at onset or had a heterozygous *PARKIN* mutation (Mori et al., 1998; Sharp et al., 2014). Patients harbouring compound heterozygous mutations of *PARKIN* where one allele was a point mutation were also more likely to have Lewy bodies than patients with homozygous exonic deletions (Doherty et al., 2013). Table 1 gives a summary of *PARKIN* PD patient autopsy data segregated by the presence or absence of Lewy body pathology. Patients without evidence of Lewy bodies were all diagnosed before the age of 40, and none had shown signs of dementia. As with most early‐onset *PARKIN* patients they were very responsive to Levodopa therapy, had a slow disease progression and did not suffer from autonomic dysfunction (Doherty & Hardy, 2013). Pathologically, all *PARKIN* patients without Lewy pathology had severe hypopigmentation of the substantia nigra and significant loss of dopaminergic neurons from this region. Several patients also had neuronal loss in the locus coeruleus, but other regions such as the amygdala, olfactory bulb, hippocampus and cortex were unaffected (Doherty & Hardy, 2013). The ascending cholinergic neurons of the pedunculopontine nucleus are frequently affected in sporadic PD (Hirsch, Graybiel, Duyckaerts, & Javoy‐Agid, 1987; Zweig, Jankel, Hedreen, Mayeux, & Price, 1989). However, this was rarely observed in *PARKIN* PD, although one case with a homozygous exon 3 *PARKIN* mutation was reported with pathology in this region (Sasaki, Shirata, Yamane, & Iwata, 2004). The focal and restricted neuronal loss in the majority of *PARKIN* patients is not typical of sporadic PD, where widespread pathology is observed that may in part be due to an α‐synuclein spreading mechanism (Desplats et al., 2009; Luk et al., 2012).

**Table 1:** Lewy body presence or absence in PD patients with *PARKIN* mutations

Sex	Age of onset	Age of death	PARKIN alleles	Lewy bodies?	Distribution of neuronal loss	Notes	References
F	NA	67	PARK2	No	Substantia nigra, locus coeruleus	Japanese, Family 556	Takahashi et al. (1994)
			PARK2				
F	20	52	PARK2	No	Substantia nigra	Japanese, Family M, patient M‐2, no dementia	Yamamura et al. (1998)
			PARK2				
M	24	62	Exon 4: deletion	No	Substantia nigra, locus coeruleus	Japanese, neurofibrillary tangles present	Mori et al. (1998)
			Exon 4: deletion				
M	32	70	Exon 4: deletion	No	Substantia nigra, locus coeruleus	Japanese, patient III‐4, no evidence of dementia	Hayashi et al. (2000)
			Exon 4: deletion				
M	18	75	Exon 3: deletion	No	Substantia nigra	Dutch, patient II‐3, tau pathology	van de Warrenburg et al. (2001)
			Exon 6: Lys211Asn				
M	34	47	Exon 2: 2‐bp deletion	No	Substantia nigra, locus coeruleus	Tunisian, patient IV‐I	Gouider‐Khouja et al. (2003)
			Exon 2: 2‐bp deletion				
F	36	86	Exon 6: deletion	No	Substantia nigra	British, no evidence of dementia	Doherty et al. (2013)
			Exon 7: Arg275Trp				
F	25	62	Exon 3: 40‐bp deletion	No	Substantia nigra	British, no evidence of dementia	Doherty et al. (2013)
			Exon 7: Arg275Trp				
M	32	68	Exon 3: 40‐bp deletion	No	Substantia nigra, locus coeruleus	Irish, no evidence of dementia	Doherty et al. (2013)
			Exon 12: Gly430Asp				
M	16	60	Intron 5: IVS5‐1G>A	No	Substantia nigra	Peruvian, patient II‐2	Cornejo‐Olivas et al. (2015)
			Exon 7: deletion				
M	20	79	Exon 3–4: deletion	No	Substantia nigra	Norwegian, no evidence of dementia	Johansen, Torp, Farrer, Gustavsson, and Aasly (2018)
			Exon 3–4: deletion				
M	41	52	Exon 3: 40‐bp deletion	Yes	Substantia nigra, locus coeruleus	North American, patient Pw3	Farrer et al. (2001)
			Exon 7: Arg275Trp				
F	33	70	Exon 3: deletion	Yes	Substantia nigra, locus coeruleus	Japanese, young onset, LBs in pedunculopontine nucleus	Sasaki et al. (2004)
			Exon 3: deletion				
M	49	73	Exon 7: deletion	Yes	Substantia nigra, locus coeruleus	Italian, patient IV.33, late onset	Pramstaller et al. (2005)
			Exon 9: 1‐bp deletion				
F	33	60	Exon 7: Arg275Trp	Yes	Substantia nigra, locus coeruleus	Irish, young onset, sparse cortical LBs	Doherty et al. (2013)
			Exon 12: Gly430Asp				
M	46	82	Exon 6: deletion	Yes	Substantia nigra, brain stem	British, no evidence of dementia, brain stem LBs	Doherty et al. (2013)
			Exon 7: Arg275Trp				
F	61	72	Exon 2–4: deletion	Yes	SN, LC, dorsal motor nucleus of the vagus, basal nucleus of Meynert	Japanese, late onset, no orthostatic hypotension or dementia	Miyakawa et al. (2013)
			Exon 2–4: deletion				

Note.

LBs: Lewy bodies; SN: substantia nigra; LC: locus coeruleus.

As dopaminergic neurons are amongst the most metabolically active of all cells in the brain (Guzman et al., 2010; Matsuda et al., 2009), functional studies of the PARKIN protein may provide a mechanism for the highly selective neuronal loss observed in this condition. Using *Drosophila* genetics, *PARKIN* was found to be downstream of *PINK1*, in a common pathway regulating mitochondrial function (Clark et al., 2006; Park et al., 2006). Extensive cell biology and biochemical analysis have uncovered the regulation of PARKIN and demonstrated that upon mitochondrial damage, it is recruited to the mitochondrial outer membrane (OMM) where it is phosphorylated and activated by PINK1 (Harper, Ordureau, & Heo, 2018; McWilliams & Muqit, 2017). Active PARKIN ubiquitylates multiple substrates at the OMM that signal the recruitment of autophagy machinery to trigger the elimination of mitochondria by autophagy (mitophagy) (Harper et al., 2018; McWilliams & Muqit, 2017). Mutations in the *PINK1* gene were identified to be the second most common cause of familial autosomal recessive PD (Valente et al., 2004). The clinical and pathological picture is similar to *PARKIN* patients with reports of patients with both Lewy and non‐Lewy pathology. A compound heterozygous *PINK1* patient who has come to autopsy did show evidence of Lewy pathology in the substantia nigra, and the nucleus basalis of Meynert, but not the locus coeruleus (Samaranch et al., 2010). This patient had a deletion of exon 7 on one allele, and a splicing mutation on the other, and it is unclear if any functional PINK1 protein was produced. In contrast, two patients with homozygous missense point mutations in PINK1 at either C388R or L347P had an absence of Lewy pathology in the substantia nigra (Steele et al., 2015; Takanashi, Li, & Hattori, 2016). More recently, PARKIN has been implicated in controlling inflammation through its role in mitophagy, and loss of PARKIN leads to increased sensitivity to stress‐induced inflammatory phenotypes that cause neurodegeneration (Sliter et al., 2018). Whilst these studies suggest a potential mechanism of neuronal death that is independent of Lewy pathology, it cannot be ruled out that α‐synuclein oligomers are formed in *PARKIN* or *PINK1* patients that contribute to neurodegeneration without forming mature Lewy bodies or Lewy neurites.

## 3 DISTINGUISHING SYNUCLEINOPATHY FROM PURE NIGROPATHY?

Here, we argue that *PARKIN* PD patients and other PD patients (e.g. *PINK1*) with predominant nigral pathology without active synucleinopathy may be ideal candidates for a cell replacement therapy for two major reasons (a) their grafts are unlikely to be affected by Lewy pathology, and (b) non‐nigral systems will remain unaffected as this is the normal course for this subtype of PD. *PARKIN* patients rarely have autonomic problems or cognitive decline, which are less likely to be addressed by a dopaminergic cell replacement therapy. The grafted neurons will also have wild‐type PARKIN protein and therefore normal mitophagy function. Due to the very focal loss of substantia nigra neurons, *PARKIN* PD is considered a pure nigropathy. However, some *PARKIN* patients also have Lewy bodies (Table 1), and although a young onset and homozygous exon deletions usually indicate a lack of Lewy pathology, at least one exception to this rule has been reported (Sasaki et al., 2004). Furthermore, a significant percentage of *LRRK2* and *PINK1* patients may also have pure nigropathy. It would therefore be valuable to stratify patients based on the absence or presence of synucleinopathy using definitive criteria.

Sympathetic denervation of the heart and Lewy pathology in the cardiac plexus is common in sporadic PD (Iwanaga et al., 1999). This can be observed non‐invasively by scintigraphy with [^123^I]metaiodobenzylguanidine (MIBG), an analogue of norepinephrine (Wieland et al., 1981). Significantly reduced cardiac MIBG uptake is associated with sporadic PD and pure autonomic failure (Braune, Reinhardt, Schnitzer, Riedel, & Lucking, 1999; Kashihara, Ohno, Kawada, & Okumura, 2006). It was found that decreased cardiac uptake of MIBG is a common feature of synucleinopathies. This method could thus be used to distinguish PD and dementia with Lewy bodies (DLB) from other neurodegenerative conditions such as progressive supranuclear palsy, and Alzheimer's disease (Orimo et al., 2007). In contrast to sporadic PD, MIBG scintigraphy of two *PARKIN* PD patients with homozygous exon 4 deletions revealed normal cardiac innervation, and they were later confirmed to have a complete absence of Lewy bodies (Orimo et al., 2005). Furthermore, a C388P *PINK1* patient that lacked Lewy pathology also had normal cardiac innervation as determined by an MIBG scan (Takanashi et al., 2016). Therefore, combining information from an MIBG scan and genetic testing for *PARKIN*,* PINK1*,* LRRK2* and other PD‐related mutations could have high predictive value for the presence or absence of synucleinopathy. However, a more definitive test for the presence or absence of synucleinopathy could be direct assaying of the cerebrospinal fluid (CSF) for the presence of α‐synuclein oligomers (Fairfoul et al., 2016; Shahnawaz et al., 2017). The RT‐QuIC assay of α‐synuclein oligomer amplification can distinguish PD and DLB from other neurodegenerative conditions, although *PARKIN* PD CSF has yet to be tested. The presence or absence of synucleinopathy can also be examined in colon, submandibular gland or skin (Del Tredici, Hawkes, Ghebremedhin, & Braak, 2010; Ikemura et al., 2008; Wakabayashi, Takahashi, Ohama, & Ikuta, 1990), and it will be interesting to investigate *PARKIN* PD at these sites for α‐synuclein pathology. Applying a combination of criteria, including clinical assessment, genetics, DAT‐SPECT imaging, MIBG scintigraphy and α‐synuclein biomarker assays, will make it possible to distinguish individuals with synucleinopathy from those with pure nigropathy caused by other mechanisms.

## 4 WHAT ABOUT SPORADIC PD?

The majority of PD patients have some form of synucleinopathy, and in some familial forms, such as *SNCA* or *GBA* mutations, it is highly active (Clark et al., 2009; Singleton et al., 2003). What could be the best approach to improve the success of a cell replacement therapy and to increase its efficacy in these patients? Fortunately, there are tremendous efforts to therapeutically target α‐synuclein disease mechanisms (Brundin, Dave, & Kordower, 2017). Passive α‐synuclein immunisation with humanised antibodies are promising and clinical trials are on‐going (Jankovic et al., 2018; Masliah et al., 2011). Clinical trials of modified anti‐sense oligonucleotides (ASOs) have been very successful for spinal muscular atrophy (Finkel et al., 2016); and a similar approach is being taken for Huntington's and Alzheimer's disease, and could be applied to Parkinson's. Progress towards reducing *SNCA* mRNA and α‐synuclein protein via RNA interference is being made (Cooper et al., 2014; Zharikov et al., 2015), and novel small molecules, such as Anle138b and NPT‐100‐18A, also have potential to reduce or eliminate α‐synuclein oligomers (Levin et al., 2014; Wrasidlo et al., 2016). Excitingly, re‐purposed drugs like salbutamol and clenbuterol, which reduce *SNCA* mRNA levels, could be used in clinical trials relatively quickly (Mittal et al., 2017). If an α‐synuclein therapeutic is successful in slowing PD, it could be used in combination with a cell replacement therapy to slow or stop the spread of Lewy pathology into the graft, as well as to other non‐nigral systems.

An alternate solution for a long‐lasting dopaminergic graft for sporadic PD patients is to provide disease‐resistant cells that are unable to form Lewy bodies. It has been described that neurons susceptible to Lewy pathology express an appreciable amount of endogenous α‐synuclein (Braak et al., 2004). Furthermore, mice that lack the *Snca* gene are completely resistant to the formation of Lewy‐like pathology triggered by stereotactic administration of recombinant α‐synuclein pre‐formed fibrils (PFFs) (Luk et al., 2012). Wild‐type mice exhibited α‐synuclein inclusions with phosphorylation at serine‐129, a hallmark of PD, 30 days after α‐synuclein PFF administration and significant dopaminergic neuronal loss by 180 days, while *Snca* ^−/−^ mice injected with PFFs did not show any signs of PD pathology or neuronal loss (Luk et al., 2012). As human pluripotent stem cells are replacing foetal tissue as a source of transplantable dopaminergic cells, it is now possible to genetically manipulate the cell product prior to transplantation. Using CRISPR/Cas9, we have deleted one or two alleles of the *SNCA* gene from human embryonic stem cells (hESCs). Upon differentiating, the modified hESCs into mDA neurons and challenging them with α‐synuclein PFFs, we demonstrated that *SNCA* ^+/−^ or *SNCA* ^−/−^ neurons exhibit partial or full resistance to the formation of Lewy‐like pathology (Chen et al., 2018). This strategy works equally well with induced pluripotent stem cells (iPSCs). An alternative approach to deleting the *SNCA* gene is to introduce a point mutation that renders the protein unable to form oligomers or fibrils, but does not affect its ability to localise to synaptic puncta or affect its normal endogenous functions. Based on in vitro fibrillation assays, and cellular aggregation assays, promising candidate residues for mutation include alanine‐76 and serine‐87 (Fiske et al., 2011; Giasson, Murray, Trojanowski, & Lee, 2001; Lázaro et al., 2014). Another route to reduce, but not eliminate, α‐synuclein expression is to over‐express natural microRNAs (miRNAs) known to target *SNCA* transcripts. Two such miRNAs are mir‐7 and mir‐153, which target the 3′ untranslated region of *SNCA* and mediates its degradation (Doxakis, 2010; Junn et al., 2009). Mir‐7 is particularly attractive as it is normally expressed in the substantia nigra and has a role in promoting cell survival (Cheng, Byrom, Shelton, & Ford, 2005; Junn et al., 2009).

## 5 CONCLUSION

Dopaminergic cell replacement therapy is rapidly approaching clinical trials for Parkinson's disease (Barker et al., 2017). The initial cell therapy efforts using foetal‐derived tissue have had mixed results for a complex variety of reasons, including patient selection (Barker et al., 2013). An unexpected outcome of the foetal trials was that the dopaminergic grafts acquired Lewy pathology and begin to fail over time (Li et al., 2016). We propose that *PARKIN* PD patients and other PD patients with pure nigropathy and lacking synucleinopathy would be ideal candidates for first‐generation dopaminergic cell replacement therapies due to their relatively focal and cell autonomous neurodegeneration. For sporadic PD, and especially PD patients with aggressive synucleinopathy, such as *GBA* PD, we advocate the use of an adjuvant therapy against α‐synuclein, when they are proven, alongside or prior to a cell therapy. Furthermore, second‐generation cell replacement therapies from hESCs or iPSCs that are genetically engineered to be resistant to Lewy pathology will increase the longevity and efficacy of the transplanted dopaminergic neurons.

## CONFLICTS OF INTEREST

MMKM serves on the Scientific Advisory Board of Amgen Inc. The other authors declare no conflicts of interest.

## AUTHORS' CONTRIBUTION

TK conceived the topic and wrote the review. AN and CC researched the literature and edited the manuscript. YC, BS, MT and SK contributed ideas and edited the manuscript. MMKM provided expert input and wrote parts of the manuscript. All authors approved the manuscript.
