# Supplementary material for: Are PARKIN patients ideal candidates for dopaminergic cell replacement therapies?
Source: Eur J Neurosci. 2019 Jan 23;49(4):453–62. doi: 10.1111/ejn.14314 (PMC6492143; doi:10.1111/ejn.14314)
Supplement: Supplementary file 1 [file EJN-49-453-s001.docx]

Are PARKIN patients ideal candidates for dopaminergic cell replacement therapies?

[Kunath, Tilo](javascript:popWindow('ejn?PARAMS=xik_BgLTd351PUfRtWQk5zsGEtPw5R7dxjL1m8iPBqwg3JqRgjatsr7Uw7JjAhAfjNU8DUDiE56fgLSv8wZEWCFS9bBpjeRDfSqGo7pjfbf3FoBVW7QyQznPt5SXnb6deh1gkNG6uAVhqGkujMojTNdPRHCgF5TkfavecR4A1QWVp9wHSGF','mailpopup_4312',%20900,%20650);) ([proxy](javascript:%20setField('XIK_CUR_ROLE_ID','');%20setField('XIK_RESESSION_USER_ID','xik_DpQXgwetyyV7F6k6RipnaiLwyFL8gVKHadKheHBEyzjV');%20setField('KEY','547772602');%20;%20setField('SWITCH_CONFIG_ID_PREV','168');%20setDataAndNextPage('XIK_PREACT',%20'xik_Grux1yopNdGDQTzxpVhqmGgsjp5WPwyLZr99o63cjJb2',%20'HOME');)) (contact); Natalwala, Ammar; Chan, Claire; Chen, Yixi; Stecher, Benjamin; Taylor, Martin; Khan, Sadaquate; Muqit, Miratul

_____________________________________________________________________________________

| Review timeline: | Submission date: 05/09/18  Editorial Decision: Minor Revision  Revision Received: 29/11/18  Accepted: 04/12/18 |  |
| --- | --- | --- |
|  |  |  |

Editor: Paul Bolam

Reviewer 1: Eilis Dowd

Reviewer 2: Sonia Gandhi

_____________________________________________________________________________________

1st Editorial Decision	23/10/18

_____________________________________________________________________________________

Are PARKIN patients ideal candidates for dopaminergic cell replacement therapies? 

Dear Dr. Kunath, 

Your manuscript has been reviewed by two external reviewers as well as by the Editorial Team. 

As you can see below, both reviewers are appreciative of your review and suggest only minor revisions.  We think that each of the points they have raised are valid but they can be addressed simply by clarification/expansion of the text.  Please carefully address each point in a revised version of your review 

We also note the following points that need to be addressed in the revised version. 
- Please supply a running title and figure/word count 
- Please supply a CoI statement, an author contributions statement and a list of abbreviations. 

When revising the manuscript, please embolden or underline major changes to the text so they are easily identifiable and please don't leave 'track change' formatting marks in your paper.  Please ensure that you provide a text and a figure file for the Graphical Abstract (as detailed in the instructions below).  When carrying out your revisions please refer to the checklist below and visit the EJN author guidelines at www.ejneuroscience.org 

When finalized, please upload your complete revised manuscript onto the website, as a Word file (.doc, or .docx).  Please also ensure that a complete set of tables and figures is included as separate files, even if these have not changed from the originals.  At this stage it is necessary to provide high resolution figures.  Please see important instructions below. 

Please go into https://mc.manuscriptcentral.com/ejn - Author Centre - manuscripts with decisions where you will find a 'create a revision' link under 'actions'. We ask that you please indicate the way in which you have responded to the points raised by the Editors and Reviewers in a letter. Please upload this response letter as a separate Word (.doc or PDF) file using the file designation "Authors' Response to Reviewers" when uploading your manuscript files.  Please DO NOT submit your revised manuscript as a new one.  Also, please note that only the  Author who submitted the original version of the manuscript should submit a revised version. 

If you are able to respond fully to the points raised, we would be pleased to receive a revision of your paper within 30 days. 

Thank you for your support of this Special Issue of EJN in memory of Tom Isaacs. 

Best wishes, 

Paul Bolam & John Foxe 
co-Editors in Chief, EJN 


Reviews: 

Reviewer: 1 

Comments to the Author 
This review/opinion piece by Tilo Kunath and colleagues proposes that, because of 1) a frequent lack of alpha-synucleinopathy/Lewy bodies and 2) a relatively focal neurodegeneration largely confined to the nigrostriatal pathway, Parkinson’s disease patients with mutations in the PARKIN gene may be ideal candidates for dopaminergic cell replacement therapy. This hypothesis largely stems from the recent report of Li et al. (2016; https://doi.org/10.1073/pnas.1605245113) who revealed that, despite 24 year maintenance of graft-derived striatal reinnervation, the patient lost graft-derived clinical benefits between 14-18 years after transplantation. This was suggested to be as a result of 1) host-to-graft transfer of synucleinopathy affecting graft functionality and 2) widespread neurodegeneration in the patient’s brain beyond the grafted region. 

Thus, the suggestion by Kunath and colleagues is interesting and reasonable, and their argument is well made. There are just a few minor aspects of the piece which could be improved as follows: 

1. The relatively focal nature of the neurodegeneration in PARKIN Parkinson’s should be introduced in the abstract as this forms a large part of the authors’ hypothesis. At present, it is just mentioned in the final sentence of the abstract, but it should be mentioned earlier. The authors do mention the motor vs. non-motor symptoms, but this would be perhaps be better framed in terms of nigral vs. non-nigral neurodegeneration. 

2. The order of studies/reports that led to the prion-like spreading hypothesis of Parkinson’s is not correct - the Luk et al. (2012) study came several years after the reports by Li et al. (2008) and Brundin et al. (2008). The authors are probably just selecting the Luk study as an example of experimental work supporting the prion-like spreading of alpha-synuclein. Nevertheless, the flow (and historical accuracy) of the paragraph would be improved if this was corrected. 

3. Typographical: Page 2. “Even before the genetic lesion was identified is was striking … “ should be “Even before the genetic lesion was identified it was striking … “. 

4. Format: Page 3. “Distinguishing synucleinopathy … “ is printing erroneously. Please check text format. 

5. Typographical: Page 3. “ … and although a young-onset … “ should be “ … and although young onset …  “ 


Reviewer: 2 

Comments to the Author 
This is a well written and articulated piece that studies an important problem in Parkinson’s disease, and presents a potential strategy. The perspective taken by the authors was unique, and well considered. 

A few changes are suggested to improve the cohesiveness of the arguments and the readability of the work, and highlight some key controversies. 

1. The abstract didn’t fully reflect the rest of the review, which essentially addresses the limitations of cell therapy due to α-synuclein spreading (and no alleviation of non motor symptoms), the use of pure nigropathy cases, biomarkers for nigropathy vs synucleinopathy, and then the approach to be used for sporadic synucleinopathy cases to prevent spread to the graft.(the last point especially is missing from abstract) 

2. Introduction: the impact and importance of a putative role for cell replacement therapy, amongst other existing or planned therapies, was not obvious (it is written as a historical account in this section). It would be clearer to set the argument for cell therapy first before patient stratification becomes relevant.   

3. Please check the numbers cited for genetic cases of PD - >10% probably needs a citation 

4. Parkin associated Parkinson’s disease – why are these cases treated differently to pink1 associated PD in the review? 

5. The authors raise the ‘potential mechanism of cell death independent of α-synuclein in parkin cases’. This is an point worth bringing out further. The distinction between synucleinopathy vs nigropathy is really a molecular pathology distinction and so perhaps the argument and terms should be used in this section (rather than only for the clinical biomarker section). If indeed, nigropathy occurs independently of synucleinopathy, then this is important to discuss in depth. I did not fully follow whether the potential mechanism in nigropathy cases was due to impaired mitophagy or the inflammation phenotypes, and what the evidence is for it being 'cell autonomous' (term used in abstract)? 

6. The potential presence of α-synuclein oligomers in parkin cases is raised – this argues against the hypothesis of synucleinopathy and nigropathy being distinct in certain cases - if oligomerisation is happening, wouldn’t this spread (and seed) to graft? This may need further clarification. 

7. The table is helpful, although the ‘notes’ are a combination of ethnicity and clinical and pathology data. A summary of the distribution of neuronal loss vs disease duration of the cases (in addition to presence of Lewy bodies) would improve the table, if the data is available. 

------------------------

____________________________________________________________________________________

Authors’ Response	29/11/18

_____________________________________________________________________________________

Dear editors, 

Thank you for the opportunity to submit a revised manuscript. 
Please find our responses to reviewer comments below. 

Best wishes, 

-Tilo 

Reviewer: 1 

Comments to the Author 
This review/opinion piece by Tilo Kunath and colleagues proposes that, because of 1) a frequent lack of alpha-synucleinopathy/Lewy bodies and 2) a relatively focal neurodegeneration largely confined to the nigrostriatal pathway, Parkinson’s disease patients with mutations in the PARKIN gene may be ideal candidates for dopaminergic cell replacement therapy. This hypothesis largely stems from the recent report of Li et al. (2016; https://doi.org/10.1073/pnas.1605245113) who revealed that, despite 24 year maintenance of graft-derived striatal reinnervation, the patient lost graft-derived clinical benefits between 14-18 years after transplantation. This was suggested to be as a result of 1) host-to-graft transfer of synucleinopathy affecting graft functionality and 2) widespread neurodegeneration in the patient’s brain beyond the grafted region. 

Response: Thank for the clear and succinct summary of our opinion piece. 

Thus, the suggestion by Kunath and colleagues is interesting and reasonable, and their argument is well made. There are just a few minor aspects of the piece which could be improved as follows: 

1. The relatively focal nature of the neurodegeneration in PARKIN Parkinson’s should be introduced in the abstract as this forms a large part of the authors’ hypothesis. At present, it is just mentioned in the final sentence of the abstract, but it should be mentioned earlier. The authors do mention the motor vs. non-motor symptoms, but this would be perhaps be better framed in terms of nigral vs. non-nigral neurodegeneration. 

Response: We completely agree the main thesis is presented too late in the abstract. This is now moved to the first half of abstract, and we have incorporated concept of non-nigral. We have also changed a sub-heading from “PARKIN Parkinson’s disease” to “Pure nigropathy Parkinson’s disease”, and broaden the discussion to include PINK1 patients, as well as selected LRRK2 cases. 

2. The order of studies/reports that led to the prion-like spreading hypothesis of Parkinson’s is not correct - the Luk et al. (2012) study came several years after the reports by Li et al. (2008) and Brundin et al. (2008). The authors are probably just selecting the Luk study as an example of experimental work supporting the prion-like spreading of alpha-synuclein. Nevertheless, the flow (and historical accuracy) of the paragraph would be improved if this was corrected. 

Response: Absolutely correct, and apologies for the reverse order it was presented. The experiment evidence is now moved later, and re-wording to show it supported the host-to-graft hypothesis published in 2008. 

3. Typographical: Page 2. “Even before the genetic lesion was identified is was striking … “ should be “Even before the genetic lesion was identified it was striking … “. 

4. Format: Page 3. “Distinguishing synucleinopathy … “ is printing erroneously. Please check text format. 

5. Typographical: Page 3. “ … and although a young-onset … “ should be “ … and although young onset … “ 

Response: Thank you, all three points are corrected. 


Reviewer: 2 

Comments to the Author 
This is a well written and articulated piece that studies an important problem in Parkinson’s disease, and presents a potential strategy. The perspective taken by the authors was unique, and well considered. 

A few changes are suggested to improve the cohesiveness of the arguments and the readability of the work, and highlight some key controversies. 

1. The abstract didn’t fully reflect the rest of the review, which essentially addresses the limitations of cell therapy due to α-synuclein spreading (and no alleviation of non motor symptoms), the use of pure nigropathy cases, biomarkers for nigropathy vs synucleinopathy, and then the approach to be used for sporadic synucleinopathy cases to prevent spread to the graft.(the last point especially is missing from abstract) 

Response: We agree this is an omission from the abstract. An additional sentence has been added to point out strategies for cell replacement therapies for sporadic Parkinson’s. 

2. Introduction: the impact and importance of a putative role for cell replacement therapy, amongst other existing or planned therapies, was not obvious (it is written as a historical account in this section). It would be clearer to set the argument for cell therapy first before patient stratification becomes relevant. 

Response: Thank you for useful suggestion. In the first paragraph of the Introduction we have referred to a recent Roger Barker review (2017) described the rapid advance to clinical of cell therapies for PD, and we refer to the recent news that the first patient was transplanted with iPSC-derived mDA cells in October 2018. 

3. Please check the numbers cited for genetic cases of PD - >10% probably needs a citation 

Response: We have cited a John Hardy review for this. 

4. Parkin associated Parkinson’s disease – why are these cases treated differently to pink1 associated PD in the review? 

Response: Thank you for pointing out this omission. We have now included a discussion of PINK1 cases. The first PINK1 autopsy reported Lewy bodies, but recent report have presented PINK1 cases that lack Lewy bodies similar to the PARKIN cases. 

5. The authors raise the ‘potential mechanism of cell death independent of α-synuclein in parkin cases’. This is an point worth bringing out further. The distinction between synucleinopathy vs nigropathy is really a molecular pathology distinction and so perhaps the argument and terms should be used in this section (rather than only for the clinical biomarker section). If indeed, nigropathy occurs independently of synucleinopathy, then this is important to discuss in depth. I did not fully follow whether the potential mechanism in nigropathy cases was due to impaired mitophagy or the inflammation phenotypes, and what the evidence is for it being 'cell autonomous' (term used in abstract)? 

Response: We have expanded the discussion of PARKIN mechanism of neuronal death significantly, and refer to 4 additional references. We also relate this mechanism to the PINK1 gene. 

6. The potential presence of α-synuclein oligomers in parkin cases is raised – this argues against the hypothesis of synucleinopathy and nigropathy being distinct in certain cases - if oligomerisation is happening, wouldn’t this spread (and seed) to graft? This may need further clarification. 

Response: At the moment, there is not evidence of aSyn oligomers in PARKIN patients. We made this statement to point to a lack of evidence. If such evidence does appear, then it is worth re-visiting the hypothesis of PARKIN PD being distinct from synucleinopathy. 


7. The table is helpful, although the ‘notes’ are a combination of ethnicity and clinical and pathology data. A summary of the distribution of neuronal loss vs disease duration of the cases (in addition to presence of Lewy bodies) would improve the table, if the data is available. 

Response: We have added an extra column for distribution of neuronal loss, and only present (i) ethinicity, (ii) patient designation (in most cases), and (iii) informative clinical/pathology comment.
